# (*R*)-Methyl {[(2-carb­oxy­bicyclo­[2.2.2]octan-1-yl)­ammonio]­methyl}­phos­phon­ate dichloro­methane 0.25-solvate

**DOI:** 10.1107/S1600536811029503

**Published:** 2011-07-30

**Authors:** Petar Todorov, Monique Calmes, Boris L. Shivachev, Rosica P. Nikolova

**Affiliations:** a Department of Organic Chemistry, University of Chemical Technology and Metallurgy, 8 Kliment Ohridski blvd, Sofia 1756, Bulgaria; b Institut des Biomolecules Max Mousseron (IBMM) UMR 5247, CNRS-Universite Montpellier 1 et 2, Universite Montpellier 2, Place E. Bataillon, 34095 Montpellier Cedex 5, France; c Institute of Mineralogy and Crystallography, Bulgarian Academy of Sciences, Acad. G. Bonchev str., bl. 107, 1113 Sofia, Bulgaria

## Abstract

The carb­oxy­lic acid mol­ecule of the title compound, C_11_H_20_NO_5_P·0.25CH_2_Cl_2_, exists as a zwitterion with the H atom of the phospho­nate group being transferred to the imine N atom. In the asymmetric unit, there are two crystallographically independent acid mol­ecules adopting the same absolute configuration and differing slightly in their geometrical parameters. In each mol­ecule, the imino and carboxyl groups are connected *via* an intra­molecular N—H⋯O hydrogen bond. Inter­molecular O—H⋯O and N—H⋯O hydrogen bonds induce the formation of layers parallel to the *ab* plane. The dichloro­methane solvent mol­ecule, with a site occupancy of 0.5, is located between the layers.

## Related literature

For general background of the use of amino­phospho­nic acid derivatives in organic synthesis and as biologically active compounds, see: Kafarski & Lejczak (2001 ▶); Orsini *et al.* (2010 ▶); Troev (2006 ▶); Naydenova *et al.* (2008 ▶, 2010 ▶).
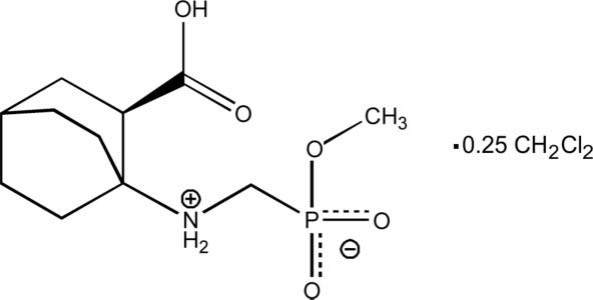


## Experimental

### 

#### Crystal data

C_11_H_20_NO_5_P·0.25CH_2_Cl_2_
*M* *_r_* = 298.48
Orthorhombic, 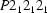

*a* = 9.3520 (2) Å
*b* = 12.7553 (3) Å
*c* = 24.1148 (8) Å
*V* = 2876.60 (13) Å^3^
*Z* = 8
Cu *K*α radiation
μ = 2.70 mm^−1^
*T* = 290 K
0.32 × 0.24 × 0.20 mm

#### Data collection

Agilent SuperNova Dual diffractometer with an Atlas detector
Absorption correction: multi-scan (*CrysAlis PRO*; Agilent, 2010 ▶) *T*_min_ = 0.151, *T*_max_ = 0.582
11759 measured reflections
4532 independent reflections
3305 reflections with *I* > 2σ(*I*)
*R*_int_ = 0.088

#### Refinement

*R*[*F* ^2^ > 2σ(*F* ^2^)] = 0.070
*wR*(*F* ^2^) = 0.205
*S* = 1.02
4532 reflections
356 parameters
H-atom parameters constrained
Δρ_max_ = 0.36 e Å^−3^
Δρ_min_ = −0.34 e Å^−3^
Absolute structure: Flack (1983 ▶), 1914 Friedel paris
Flack parameter: 0.04 (4)

### 

Data collection: *CrysAlis PRO* (Agilent, 2010 ▶); cell refinement: *CrysAlis PRO*; data reduction: *CrysAlis PRO*; program(s) used to solve structure: *SHELXS97* (Sheldrick, 2008 ▶); program(s) used to refine structure: *SHELXL97* (Sheldrick, 2008 ▶); molecular graphics: *ORTEP-3 for Windows* (Farrugia, 1997 ▶); software used to prepare material for publication: *WinGX* (Farrugia, 1999 ▶) and *Mercury* (Macrae *et al.*, 2008 ▶).

## Supplementary Material

Crystal structure: contains datablock(s) I, global. DOI: 10.1107/S1600536811029503/is2751sup1.cif

Structure factors: contains datablock(s) I. DOI: 10.1107/S1600536811029503/is2751Isup2.hkl

Supplementary material file. DOI: 10.1107/S1600536811029503/is2751Isup3.cml

Additional supplementary materials:  crystallographic information; 3D view; checkCIF report

## Figures and Tables

**Table 1:** Hydrogen-bond geometry (Å, °)

*D*—H⋯*A*	*D*—H	H⋯*A*	*D*⋯*A*	*D*—H⋯*A*
N1—H1*A*⋯O24^i^	0.90	1.79	2.673 (6)	168
N1—H1*B*⋯O1	0.90	1.93	2.683 (6)	140
N21—H21*A*⋯O22	0.90	2.04	2.750 (7)	135
N21—H21*B*⋯O5^ii^	0.90	1.79	2.690 (6)	173
O2—H2⋯O4^ii^	0.82	1.66	2.474 (6)	172
O22—H22⋯O25^iii^	0.82	1.86	2.596 (6)	149

Symmetry codes: (i) 
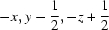
; (ii) 
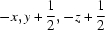
; (iii) 
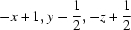
.

**Fig. 1.**
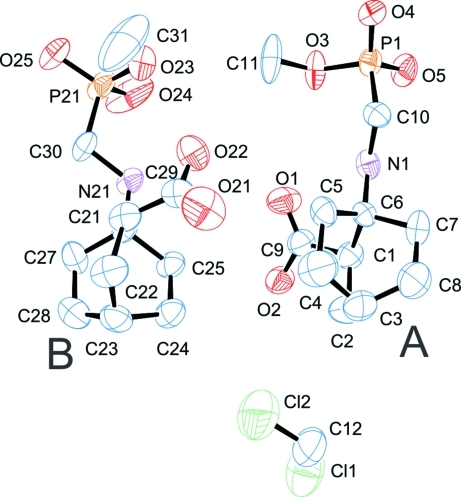
The asymmetric unit of title compound with the atom numbering scheme showing 50% probability displacement ellipsoids. H atoms have been omitted for clarity.

**Fig. 2.**
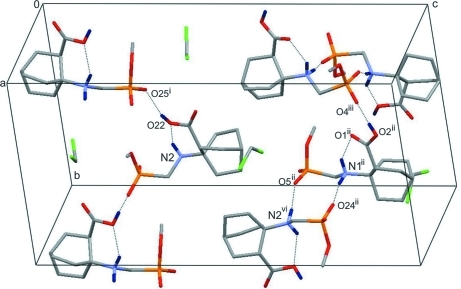
The packing arrangement of the molecules in the unit cell, showing the hydrogen-bonding interactions as dashed lines. [symmetry codes: (i) 1 - x, y - 1/2, 1/2 - z; (ii) 1/2 - x, 1 - y, 1/2 + z; (iii) 1/2 + x, 1/2 - y, 1 - z; (iv) 1/2 + x, 1.5 - y, 1 - z;

**Fig. 3.**
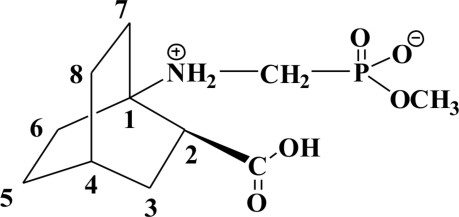
NMR numbering scheme.

## supplementary crystallographic information

### Comment

α-Aminophosphonic acids occupy an important place amongst the various compounds containing a P—C bond and an amino group, because they are analogues of natural α-amino acids, the building blocks of peptides and proteins. Since stereochemistry at the α-carbon atom plays an important role in the biological activity of the molecule, the synthesis of chiral α-aminophosphonates and α-aminophosphonic acids has been a focus of considerable attention in synthetic organic chemistry as well as in modern pharmaceutical chemistry. This is underlined by an increasing number of industrial applications in the field of the synthesis of *enantio* enriched α-aminophosphonic acid derivatives (Kafarski & Lejczak, 2001; Troev, 2006; Naydenova *et al.*, 2010). This, together with their low mammalian toxicity makes the α-aminophosphonic acids an important class of antimetabolites and a potential source of medicinal lead compounds (Naydenova *et al.*, 2008; Orsini *et al.*, 2010).

The title compound has been obtained in an enantiopure form. Herein, we synthesized and characterized a new α-aminophosphonate containing bicyclo[2.2.2]octane-moiety by Kabachnik-Fields reaction. This reaction is performed without epimerization. The optically active (*R*)-1-(*N*-(methoxyphosphonomethyl)aminobicyclo [2.2.2]octane-2-carboxylic acid was purified by column chromatography on silica gel using a mixture of dichloromethane/methanol (9.5/0.5) with 0.1% acetic acid as eluent to give the title compound.

The studied compound C_11_H_10_NPO_5_ crystallizes as a dichloromethane 0.5 solvate with two crystallographically nonequivalent molecules (molecules A and B in Fig. 1). NMR analyses (^1^H, ^13^C and ^31^P) has been applied for compositional and geometrical characterization of the organic molecule. The ^1^H-NMR data were not indicative for the presence of NH2 or P—OH groups. From the other hand the difference Fourier analyses around imine N and PO_3_ group show presence of two hydrogen atoms around the nitrogen. More over the P—O distances of 1.491 (4), 1.494 (4) Å and 1.459 (5), 1.481 (4) Å for molecules A and B, respectively, suppose charge distribution between the phosphonate O atoms. This suggests that both molecules exist as zwitterions with H atom of the phosphonate group being transferred to the imine N atom. The absolute configuration of the independent C_11_H_10_NPO_5_ molecules is identical as deduced by the Flack parameter. The rotation of the bicyclo[2.2.2]octane-2-carboxylic fragment along axis described by N1,C6, C3 and N21, C26, C23 atoms for A and B molecules respectively differ by less than 10° [57.6 (2)° for A and 64.7 (1) ° for B molecule].

The imino and carboxylic groups are involved in intramolecular hydrogen bond in both of the molecules. The O—H···O and N—H···O intermolecular interactions induce the formation of layers parallel to the crystallographic *ab* plane. The dichloromethane moieties are located in the cavities near by the zigzag layer.

### Experimental

Paraformaldehyde (1.827 mmol), methanol (5 ml), and triethylamine (190 υl) were put into a three-necked flask equipped with a condenser, magnetic stirrer, thermometer and dropping funnel and argon inert. The reaction mixture was heated to reflux temperature and held there for 45 min, after which it became a clear solution. (*R*)-1-Aminobicyclo[2.2.2]octane-2-carboxylic acid (1.175 mmol) and triethylamine (0.24 ml) were added to this solution. The suspension was heated at 65 - 70 °C and after 3.5 h it became a clear solution. Dimethyl hydrogen phosphonate 122 µl (146.5 mg, 1.331 mmol) was added to this solution for approximately 10 min. This reaction mixture was heated at 65–70 °C and after maintaining this temperature for 5.5 h, it was cooled to room temperature and concentrated under reduced pressure. The crude compound was dissolved in methanol and the non-reacting (*R*)-1-aminobicyclo[2.2.2]octane-2-carboxylic acid was removed by precipitation with diethyl ether and collected by filtration. The filtrate was evaporated to give a residue which was purified by flash column chromatography on silica gel using a mixture of dichloromethane/methanol (9.5/0.5) with 0.1% acetic acid as eluent to yield the 1-(*N*-methoxyphosphonomethyl)aminobicyclo[2.2.2]octane- 2-carboxylic acid.

^1^H NMR (400 MHz, CDCl_3_), δ = 1.19–1.75 (m, 9 H, 4-H, 5-H, 6-H, 8-H, 3-H, 6-H), 2.34 (br.d, *J* = 12.9 Hz, 1H, 7-H), 2.48 (br.d, *J* = 10.9 Hz, 1H, 2-H), 2.92 (AB part of ABX system, 2H, ^2^*J*P—H = 15.0 Hz, P—CH_2_), 3.85 (d, 6H, ^3^*J*P—H = 10.9 Hz, O—CH_3_), 7.45 (br.s, 1H, COOH).

^13^C NMR (100 MHz, CD_3_OD), δ = 25.25 (C-4), 26.41, 26.79, 26.87, 28.90 and 30.09 (CH_2_), 43.10 (C-2), 57.29 (C-1), 35.10 (d, ^2^1*J*P—C = 156.0 Hz, P—CH_2_), 54.14 (d, ^2^*J*P—C = 10.6 Hz, O—CH_3_), 179.66 (C=O).

^31^P NMR (161.97 MHz, CDCl_3_),δ = 25.19.

For NMR numbering see Fig.3

### Refinement

All H atoms bonded to C, N and O were placed in idealized positions (C—H_methyl_ = 0.96 Å, C—H_methylene_ = 0.97 Å, C—H_methyne_ = 0.98 Å, N—H = 0.86 Å and O—H = 0.82 Å) and were constrained to ride on their parent atoms, with *U*_iso_(H) = 1.2*U*_eq_(C or N) or 1.5*U*_eq_(O or C_methyl_).

### Crystal data

**Table:**

C_11_H_20_NO_5_P·0.25CH_2_Cl_2_	*F*(000) = 1268
*M**_r_* = 298.48	*D*_x_ = 1.378 Mg m^−^^3^
Orthorhombic, *P*2_1_2_1_2_1_	Cu *K*α radiation, λ = 1.5418 Å
Hall symbol: P 2ac 2ab	Cell parameters from 5494 reflections
*a* = 9.3520 (2) Å	θ = 3.5–62.6°
*b* = 12.7553 (3) Å	µ = 2.70 mm^−^^1^
*c* = 24.1148 (8) Å	*T* = 290 K
*V* = 2876.60 (13) Å^3^	Prism, colorless
*Z* = 8	0.32 × 0.24 × 0.20 mm

### Data collection

**Table:**

Agilent SuperNova Dual diffractometer with an Atlas detector	4532 independent reflections
Radiation source: SuperNova (Cu) X-ray Source	3305 reflections with *I* > 2σ(*I*)
mirror	*R*_int_ = 0.088
Detector resolution: 10.3974 pixels mm^-1^	θ_max_ = 62.6°, θ_min_ = 3.7°
ω scans	*h* = −10→10
Absorption correction: multi-scan (*CrysAlis PRO*; Agilent, 2010)	*k* = −14→14
*T*_min_ = 0.151, *T*_max_ = 0.582	*l* = −27→26
11759 measured reflections	

### Refinement

**Table:**

Refinement on *F*^2^	Secondary atom site location: difference Fourier map
Least-squares matrix: full	Hydrogen site location: inferred from neighbouring sites
*R*[*F*^2^ > 2σ(*F*^2^)] = 0.070	H-atom parameters constrained
*wR*(*F*^2^) = 0.205	*w* = 1/[σ^2^(*F*_o_^2^) + (0.1029*P*)^2^ + 1.5603*P*] where *P* = (*F*_o_^2^ + 2*F*_c_^2^)/3
*S* = 1.02	(Δ/σ)_max_ < 0.001
4532 reflections	Δρ_max_ = 0.36 e Å^−^^3^
356 parameters	Δρ_min_ = −0.34 e Å^−^^3^
0 restraints	Absolute structure: Flack (1983), 1914 Friedel paris
Primary atom site location: structure-invariant direct methods	Flack parameter: 0.04 (4)

### Special details

**Table:**

Geometry. All e.s.d.'s (except the e.s.d. in the dihedral angle between two l.s. planes) are estimated using the full covariance matrix. The cell e.s.d.'s are taken into account individually in the estimation of e.s.d.'s in distances, angles and torsion angles; correlations between e.s.d.'s in cell parameters are only used when they are defined by crystal symmetry. An approximate (isotropic) treatment of cell e.s.d.'s is used for estimating e.s.d.'s involving l.s. planes.
Refinement. Refinement of *F*^2^ against ALL reflections. The weighted *R*-factor *wR* and goodness of fit *S* are based on *F*^2^, conventional *R*-factors *R* are based on *F*, with *F* set to zero for negative *F*^2^. The threshold expression of *F*^2^ > σ(*F*^2^) is used only for calculating *R*-factors(gt) *etc*. and is not relevant to the choice of reflections for refinement. *R*-factors based on *F*^2^ are statistically about twice as large as those based on *F*, and *R*- factors based on ALL data will be even larger.

### Fractional atomic coordinates and isotropic or equivalent isotropic displacement parameters (Å^2^)

**Table:**

	*x*	*y*	*z*	*U*_iso_*/*U*_eq_	Occ. (<1)
C1	−0.1337 (7)	0.3754 (5)	0.3764 (3)	0.0544 (16)	
H1	−0.2314	0.3609	0.3639	0.065*	
C2	−0.1299 (10)	0.3581 (7)	0.4400 (3)	0.088 (3)	
H2A	−0.1011	0.4225	0.4582	0.106*	
H2B	−0.2247	0.3397	0.4531	0.106*	
C3	−0.0267 (9)	0.2721 (6)	0.4544 (3)	0.078 (2)	
H3	−0.0215	0.2620	0.4946	0.094*	
C4	0.1183 (8)	0.3043 (10)	0.4311 (4)	0.104 (3)	
H4A	0.1903	0.2549	0.4436	0.124*	
H4B	0.1435	0.3729	0.4455	0.124*	
C5	0.1193 (6)	0.3083 (5)	0.3675 (3)	0.0546 (15)	
H5A	0.1567	0.3751	0.3549	0.066*	
H5B	0.1791	0.2528	0.3528	0.066*	
C6	−0.0345 (6)	0.2944 (4)	0.3481 (2)	0.0427 (12)	
C7	−0.0851 (7)	0.1847 (5)	0.3641 (3)	0.0623 (18)	
H7A	−0.1841	0.1754	0.3531	0.075*	
H7B	−0.0279	0.1324	0.3451	0.075*	
C8	−0.0712 (12)	0.1713 (6)	0.4255 (4)	0.102 (3)	
H8A	−0.0008	0.1173	0.4331	0.123*	
H8B	−0.1620	0.1480	0.4405	0.123*	
C9	−0.0977 (7)	0.4880 (5)	0.3593 (3)	0.0566 (16)	
C10	0.0450 (6)	0.2480 (5)	0.2480 (2)	0.0478 (14)	
H10A	0.0197	0.1743	0.2499	0.057*	
H10B	0.1438	0.2551	0.2596	0.057*	
C11	0.2109 (7)	0.4460 (6)	0.1910 (4)	0.093 (3)	
H11A	0.2119	0.5114	0.2107	0.139*	
H11B	0.2574	0.4546	0.1558	0.139*	
H11C	0.2604	0.3938	0.2123	0.139*	
C12	−0.098 (2)	0.3952 (18)	0.5960 (10)	0.134 (9)	0.50
H12A	−0.0840	0.3587	0.6309	0.161*	0.50
H12B	−0.1257	0.3432	0.5688	0.161*	0.50
C21	0.5079 (6)	0.5691 (5)	0.4133 (3)	0.0538 (16)	
H21	0.5952	0.5949	0.3954	0.065*	
C22	0.5372 (7)	0.5643 (7)	0.4775 (3)	0.072 (2)	
H22A	0.5465	0.4918	0.4891	0.086*	
H22B	0.6261	0.6001	0.4859	0.086*	
C23	0.4134 (8)	0.6165 (6)	0.5092 (3)	0.0646 (18)	
H23	0.4219	0.6029	0.5490	0.078*	
C24	0.2722 (7)	0.5731 (6)	0.4870 (3)	0.0608 (17)	
H24A	0.2734	0.4971	0.4888	0.073*	
H24B	0.1937	0.5981	0.5097	0.073*	
C25	0.2507 (6)	0.6079 (5)	0.4276 (3)	0.0523 (16)	
H25A	0.1764	0.6608	0.4260	0.063*	
H25B	0.2205	0.5487	0.4052	0.063*	
C26	0.3902 (5)	0.6526 (4)	0.4044 (2)	0.0422 (13)	
C27	0.4266 (8)	0.7527 (5)	0.4352 (3)	0.0643 (18)	
H27A	0.3602	0.8078	0.4249	0.077*	
H27B	0.5224	0.7754	0.4255	0.077*	
C28	0.4176 (10)	0.7321 (6)	0.4980 (3)	0.082 (2)	
H28A	0.4999	0.7628	0.5163	0.098*	
H28B	0.3322	0.7649	0.5128	0.098*	
C29	0.4765 (7)	0.4605 (5)	0.3898 (3)	0.0581 (16)	
C30	0.4830 (6)	0.7393 (5)	0.3151 (2)	0.0523 (15)	
H30A	0.5762	0.7141	0.3268	0.063*	
H30B	0.4748	0.8120	0.3265	0.063*	
C31	0.6373 (13)	0.5596 (10)	0.2371 (8)	0.196 (8)	
H31A	0.6546	0.5112	0.2073	0.293*	
H31B	0.6415	0.5231	0.2719	0.293*	
H31C	0.7087	0.6136	0.2365	0.293*	
N1	−0.0493 (5)	0.3092 (4)	0.28655 (19)	0.0476 (11)	
H1A	−0.1406	0.2946	0.2775	0.057*	
H1B	−0.0353	0.3777	0.2793	0.057*	
N21	0.3695 (4)	0.6765 (4)	0.3434 (2)	0.0475 (12)	
H21A	0.3609	0.6151	0.3253	0.057*	
H21B	0.2860	0.7108	0.3396	0.057*	
O1	−0.0342 (6)	0.5114 (3)	0.3167 (2)	0.0776 (15)	
O2	−0.1409 (5)	0.5589 (3)	0.3936 (2)	0.0646 (13)	
H2	−0.1349	0.6168	0.3790	0.097*	
O3	0.0658 (5)	0.4134 (3)	0.1823 (2)	0.0641 (13)	
O4	0.1387 (5)	0.2392 (3)	0.14410 (18)	0.0602 (12)	
O5	−0.1266 (4)	0.2891 (4)	0.16076 (19)	0.0639 (12)	
O21	0.4858 (7)	0.3804 (4)	0.4138 (3)	0.100 (2)	
O22	0.4354 (6)	0.4666 (4)	0.3379 (2)	0.0729 (14)	
H22	0.4600	0.4135	0.3213	0.109*	
O23	0.4943 (7)	0.6072 (5)	0.2303 (2)	0.107 (2)	
O24	0.3242 (5)	0.7561 (7)	0.2252 (2)	0.128 (3)	
O25	0.5913 (4)	0.7934 (4)	0.21767 (19)	0.0665 (13)	
P1	0.02660 (16)	0.29346 (12)	0.17764 (7)	0.0495 (4)	
P21	0.47115 (16)	0.73176 (16)	0.24091 (7)	0.0615 (5)	
Cl1	−0.2400 (7)	0.4823 (5)	0.6043 (3)	0.139 (2)	0.50
Cl2	0.0552 (7)	0.4434 (7)	0.5772 (3)	0.159 (3)	0.50

### Atomic displacement parameters (Å^2^)

**Table:**

	*U*^11^	*U*^22^	*U*^33^	*U*^12^	*U*^13^	*U*^23^
C1	0.049 (4)	0.055 (4)	0.059 (4)	0.001 (3)	0.004 (3)	0.002 (3)
C2	0.110 (7)	0.090 (6)	0.065 (6)	0.019 (5)	0.022 (5)	0.010 (4)
C3	0.097 (6)	0.087 (5)	0.050 (4)	−0.016 (5)	−0.004 (4)	0.016 (4)
C4	0.062 (5)	0.172 (10)	0.077 (6)	−0.024 (6)	−0.017 (4)	0.001 (6)
C5	0.044 (3)	0.062 (4)	0.058 (4)	−0.004 (3)	−0.012 (3)	−0.005 (3)
C6	0.042 (3)	0.042 (3)	0.044 (3)	0.002 (3)	−0.004 (2)	−0.004 (2)
C7	0.058 (4)	0.046 (3)	0.083 (5)	−0.014 (3)	−0.006 (3)	0.007 (3)
C8	0.164 (9)	0.064 (5)	0.080 (6)	−0.025 (6)	0.006 (6)	0.021 (4)
C9	0.058 (4)	0.052 (4)	0.059 (4)	0.002 (3)	0.008 (3)	0.002 (3)
C10	0.038 (3)	0.055 (3)	0.051 (4)	0.006 (3)	−0.001 (2)	−0.007 (3)
C11	0.044 (4)	0.065 (4)	0.170 (9)	−0.025 (4)	−0.034 (5)	0.014 (5)
C12	0.131 (18)	0.131 (18)	0.14 (2)	−0.060 (16)	0.064 (15)	−0.039 (15)
C21	0.030 (3)	0.076 (4)	0.055 (4)	0.013 (3)	−0.004 (3)	0.005 (3)
C22	0.047 (4)	0.106 (6)	0.063 (5)	0.012 (4)	−0.007 (3)	0.013 (4)
C23	0.074 (5)	0.078 (4)	0.042 (4)	0.000 (4)	−0.008 (3)	0.001 (3)
C24	0.048 (4)	0.073 (4)	0.062 (5)	−0.002 (3)	0.004 (3)	0.006 (4)
C25	0.036 (3)	0.065 (4)	0.055 (4)	0.002 (3)	0.007 (3)	0.001 (3)
C26	0.029 (3)	0.051 (3)	0.047 (4)	0.002 (2)	0.000 (2)	0.006 (3)
C27	0.071 (4)	0.056 (4)	0.066 (5)	−0.011 (3)	−0.014 (3)	−0.008 (3)
C28	0.101 (6)	0.079 (5)	0.066 (5)	−0.010 (4)	0.000 (4)	−0.017 (4)
C29	0.052 (3)	0.072 (4)	0.051 (4)	0.009 (3)	−0.004 (3)	0.004 (3)
C30	0.034 (3)	0.064 (4)	0.059 (4)	−0.012 (3)	−0.003 (3)	0.020 (3)
C31	0.118 (10)	0.106 (8)	0.36 (2)	0.020 (8)	0.138 (13)	0.024 (11)
N1	0.035 (2)	0.049 (3)	0.059 (3)	0.000 (2)	−0.013 (2)	−0.004 (2)
N21	0.023 (2)	0.069 (3)	0.051 (3)	0.001 (2)	−0.0021 (19)	0.007 (2)
O1	0.113 (4)	0.047 (2)	0.073 (3)	0.015 (3)	0.032 (3)	0.001 (2)
O2	0.066 (3)	0.053 (2)	0.076 (3)	0.004 (2)	0.024 (2)	−0.005 (2)
O3	0.054 (3)	0.036 (2)	0.102 (4)	−0.0116 (18)	−0.023 (2)	0.013 (2)
O4	0.061 (3)	0.062 (2)	0.058 (3)	−0.009 (2)	0.017 (2)	−0.003 (2)
O5	0.044 (2)	0.081 (3)	0.067 (3)	−0.018 (2)	−0.019 (2)	0.008 (2)
O21	0.142 (6)	0.057 (3)	0.102 (4)	0.026 (3)	−0.012 (4)	0.014 (3)
O22	0.080 (3)	0.059 (3)	0.080 (4)	0.005 (3)	−0.008 (3)	−0.008 (2)
O23	0.115 (5)	0.105 (4)	0.101 (5)	−0.058 (4)	0.028 (4)	−0.022 (3)
O24	0.030 (2)	0.279 (10)	0.073 (4)	0.000 (4)	−0.013 (2)	0.060 (5)
O25	0.042 (2)	0.086 (3)	0.071 (3)	−0.007 (2)	0.007 (2)	0.030 (2)
P1	0.0394 (7)	0.0521 (8)	0.0569 (10)	−0.0083 (7)	−0.0040 (7)	0.0004 (7)
P21	0.0294 (7)	0.0956 (13)	0.0596 (11)	−0.0111 (9)	−0.0006 (7)	0.0131 (9)
Cl1	0.115 (4)	0.117 (4)	0.185 (7)	−0.017 (4)	−0.031 (4)	0.044 (4)
Cl2	0.107 (4)	0.199 (7)	0.171 (6)	−0.050 (5)	0.002 (4)	−0.003 (5)

### Geometric parameters (Å, °)

**Table:**

C1—C9	1.532 (9)	C22—C23	1.539 (10)
C1—C6	1.546 (8)	C22—H22A	0.9700
C1—C2	1.550 (10)	C22—H22B	0.9700
C1—H1	0.9800	C23—C28	1.500 (10)
C2—C3	1.502 (11)	C23—C24	1.528 (9)
C2—H2A	0.9700	C23—H23	0.9800
C2—H2B	0.9700	C24—C25	1.515 (9)
C3—C8	1.521 (11)	C24—H24A	0.9700
C3—C4	1.524 (11)	C24—H24B	0.9700
C3—H3	0.9800	C25—C26	1.529 (7)
C4—C5	1.533 (10)	C25—H25A	0.9700
C4—H4A	0.9700	C25—H25B	0.9700
C4—H4B	0.9700	C26—N21	1.515 (7)
C5—C6	1.523 (7)	C26—C27	1.516 (8)
C5—H5A	0.9700	C27—C28	1.537 (10)
C5—H5B	0.9700	C27—H27A	0.9700
C6—N1	1.503 (7)	C27—H27B	0.9700
C6—C7	1.527 (8)	C28—H28A	0.9700
C7—C8	1.495 (10)	C28—H28B	0.9700
C7—H7A	0.9700	C29—O21	1.177 (8)
C7—H7B	0.9700	C29—O22	1.313 (7)
C8—H8A	0.9700	C30—N21	1.495 (7)
C8—H8B	0.9700	C30—P21	1.795 (6)
C9—O1	1.223 (7)	C30—H30A	0.9700
C9—O2	1.291 (7)	C30—H30B	0.9700
C10—N1	1.501 (7)	C31—O23	1.478 (13)
C10—P1	1.802 (6)	C31—H31A	0.9600
C10—H10A	0.9700	C31—H31B	0.9600
C10—H10B	0.9700	C31—H31C	0.9600
C11—O3	1.434 (7)	N1—H1A	0.9000
C11—H11A	0.9600	N1—H1B	0.9000
C11—H11B	0.9600	N21—H21A	0.9000
C11—H11C	0.9600	N21—H21B	0.9000
C12—Cl2	1.622 (19)	O2—H2	0.8200
C12—Cl1	1.74 (2)	O3—P1	1.578 (4)
C12—H12A	0.9700	O4—P1	1.494 (4)
C12—H12B	0.9700	O5—P1	1.491 (4)
C21—C29	1.525 (9)	O22—H22	0.8200
C21—C26	1.547 (8)	O23—P21	1.624 (7)
C21—C22	1.572 (9)	O24—P21	1.459 (5)
C21—H21	0.9800	O25—P21	1.481 (4)
			
C9—C1—C6	112.1 (5)	H22A—C22—H22B	108.2
C9—C1—C2	113.2 (6)	C28—C23—C24	108.5 (6)
C6—C1—C2	109.1 (5)	C28—C23—C22	108.5 (7)
C9—C1—H1	107.4	C24—C23—C22	108.6 (6)
C6—C1—H1	107.4	C28—C23—H23	110.4
C2—C1—H1	107.4	C24—C23—H23	110.4
C3—C2—C1	110.3 (6)	C22—C23—H23	110.4
C3—C2—H2A	109.6	C25—C24—C23	109.8 (5)
C1—C2—H2A	109.6	C25—C24—H24A	109.7
C3—C2—H2B	109.6	C23—C24—H24A	109.7
C1—C2—H2B	109.6	C25—C24—H24B	109.7
H2A—C2—H2B	108.1	C23—C24—H24B	109.7
C2—C3—C8	109.6 (7)	H24A—C24—H24B	108.2
C2—C3—C4	106.8 (7)	C24—C25—C26	110.0 (5)
C8—C3—C4	107.6 (8)	C24—C25—H25A	109.7
C2—C3—H3	110.9	C26—C25—H25A	109.7
C8—C3—H3	110.9	C24—C25—H25B	109.7
C4—C3—H3	110.9	C26—C25—H25B	109.7
C3—C4—C5	112.5 (6)	H25A—C25—H25B	108.2
C3—C4—H4A	109.1	N21—C26—C27	109.6 (5)
C5—C4—H4A	109.1	N21—C26—C25	108.7 (4)
C3—C4—H4B	109.1	C27—C26—C25	109.1 (5)
C5—C4—H4B	109.1	N21—C26—C21	111.3 (5)
H4A—C4—H4B	107.8	C27—C26—C21	110.6 (5)
C6—C5—C4	107.4 (5)	C25—C26—C21	107.4 (5)
C6—C5—H5A	110.2	C26—C27—C28	109.0 (6)
C4—C5—H5A	110.2	C26—C27—H27A	109.9
C6—C5—H5B	110.2	C28—C27—H27A	109.9
C4—C5—H5B	110.2	C26—C27—H27B	109.9
H5A—C5—H5B	108.5	C28—C27—H27B	109.9
N1—C6—C5	112.1 (5)	H27A—C27—H27B	108.3
N1—C6—C7	109.6 (5)	C23—C28—C27	110.3 (6)
C5—C6—C7	108.8 (5)	C23—C28—H28A	109.6
N1—C6—C1	107.2 (4)	C27—C28—H28A	109.6
C5—C6—C1	110.7 (5)	C23—C28—H28B	109.6
C7—C6—C1	108.3 (5)	C27—C28—H28B	109.6
C8—C7—C6	109.1 (6)	H28A—C28—H28B	108.1
C8—C7—H7A	109.9	O21—C29—O22	122.9 (7)
C6—C7—H7A	109.9	O21—C29—C21	126.2 (6)
C8—C7—H7B	109.9	O22—C29—C21	110.9 (5)
C6—C7—H7B	109.9	N21—C30—P21	112.5 (4)
H7A—C7—H7B	108.3	N21—C30—H30A	109.1
C7—C8—C3	112.4 (6)	P21—C30—H30A	109.1
C7—C8—H8A	109.1	N21—C30—H30B	109.1
C3—C8—H8A	109.1	P21—C30—H30B	109.1
C7—C8—H8B	109.1	H30A—C30—H30B	107.8
C3—C8—H8B	109.1	O23—C31—H31A	109.5
H8A—C8—H8B	107.9	O23—C31—H31B	109.5
O1—C9—O2	121.3 (6)	H31A—C31—H31B	109.5
O1—C9—C1	124.1 (6)	O23—C31—H31C	109.5
O2—C9—C1	114.6 (6)	H31A—C31—H31C	109.5
N1—C10—P1	111.1 (4)	H31B—C31—H31C	109.5
N1—C10—H10A	109.4	C10—N1—C6	119.5 (4)
P1—C10—H10A	109.4	C10—N1—H1A	107.5
N1—C10—H10B	109.4	C6—N1—H1A	107.5
P1—C10—H10B	109.4	C10—N1—H1B	107.5
H10A—C10—H10B	108.0	C6—N1—H1B	107.5
O3—C11—H11A	109.5	H1A—N1—H1B	107.0
O3—C11—H11B	109.5	C30—N21—C26	117.4 (4)
H11A—C11—H11B	109.5	C30—N21—H21A	107.9
O3—C11—H11C	109.5	C26—N21—H21A	107.9
H11A—C11—H11C	109.5	C30—N21—H21B	107.9
H11B—C11—H11C	109.5	C26—N21—H21B	107.9
Cl2—C12—Cl1	117.6 (14)	H21A—N21—H21B	107.2
Cl2—C12—H12A	107.9	C9—O2—H2	109.5
Cl1—C12—H12A	107.9	C11—O3—P1	120.8 (4)
Cl2—C12—H12B	107.9	C29—O22—H22	109.5
Cl1—C12—H12B	107.9	C31—O23—P21	120.3 (6)
H12A—C12—H12B	107.2	O5—P1—O4	120.6 (3)
C29—C21—C26	115.9 (5)	O5—P1—O3	106.2 (3)
C29—C21—C22	111.3 (5)	O4—P1—O3	108.9 (3)
C26—C21—C22	106.7 (5)	O5—P1—C10	109.7 (3)
C29—C21—H21	107.5	O4—P1—C10	107.1 (3)
C26—C21—H21	107.5	O3—P1—C10	102.9 (3)
C22—C21—H21	107.5	O24—P21—O25	120.2 (3)
C23—C22—C21	109.9 (5)	O24—P21—O23	107.0 (4)
C23—C22—H22A	109.7	O25—P21—O23	111.0 (3)
C21—C22—H22A	109.7	O24—P21—C30	107.8 (3)
C23—C22—H22B	109.7	O25—P21—C30	107.6 (3)
C21—C22—H22B	109.7	O23—P21—C30	101.6 (3)

### Hydrogen-bond geometry (Å, °)

**Table:**

*D*—H···*A*	*D*—H	H···*A*	*D*···*A*	*D*—H···*A*
N1—H1A···O24^i^	0.90	1.79	2.673 (6)	168.
N1—H1B···O1	0.90	1.93	2.683 (6)	140.
N21—H21A···O22	0.90	2.04	2.750 (7)	135.
N21—H21B···O5^ii^	0.90	1.79	2.690 (6)	173.
O2—H2···O4^ii^	0.82	1.66	2.474 (6)	172.
O22—H22···O25^iii^	0.82	1.86	2.596 (6)	149.

Symmetry codes: (i) −*x*, *y*−1/2, −*z*+1/2; (ii) −*x*, *y*+1/2, −*z*+1/2; (iii) −*x*+1, *y*−1/2, −*z*+1/2.
